# Editorial: Dry eye disease syndrome

**DOI:** 10.3389/fmed.2022.1104593

**Published:** 2023-01-04

**Authors:** Alejandro Navas, Enrique O. Graue-Hernández, Victor L. Perez, Yonathan Garfias

**Affiliations:** ^1^Cell and Tissue Biology and Cornea Departments, Instituto de Oftalmología Fundación de Asistencia Privada Conde de Valenciana, I.A.P, Mexico City, Mexico; ^2^Department of Ophthalmology, School of Medicine, Duke University, Durham, NC, United States; ^3^Department of Biochemistry, Faculty of Medicine, National Autonomous University of Mexico, Mexico City, Mexico

**Keywords:** dry eye disease, epidemiology, pathophysiology, etiology, treatment, diagnosis

Dry eye is a complex multifactorial disease with significant morbidity and effects on quality of life. In this Research Topic, the reader will find important advances in the understanding of its pathophysiology, epidemiology, diagnosis, and novel treatments.

Beginning with an exquisite perspective by Soifer et al., presenting in a narrative review proposing a subcategory of ocular surface inflammatory disorders (OSIDs) for dry eye disease in which the role of inflammation, immune phenotypes and anti-inflammatory therapies is very well-described. The reader will also find a review performed by Asiedu regarding some molecular compounds as potential indicators for meibomian gland dysfunction (MGD). This review of over 100 articles summarizes the main lipids, proteins, carbohydrates, amino acids and enzymes with potential relationships as biomarkers for MGD. DED is more prevalent in women than in men, thus, a hormonal status has been associated with DED. Jüngert et al. described for the first time the presence of prolactin (PRL), its receptor (PRLR) and the prolactin inducible peptide (PIP) in the human lacrimal apparatus and in the ocular surface. They showed that PIP levels are increased in reflex tears of DED patients with mixed DED. This research opens the possibility of measuring PIP as a biomarker in DED. As is well-known, DED is accompanied by an inflammatory response. To downregulate the inflammatory environment, Yu et al. tested small molecules functioning as tyrosine kinase (TrK) receptor agonists in a mouse DED model. Interestingly, they found that TrK receptor agonists improve DED at inhibiting NFkB activation and upregulating TNFAIP3 and EP4 anti-inflammatory proteins. This research emphasizes the necessity to find novel efficacious molecules to accurately treat DED by selectively inhibiting the inflammatory environment.

These very interesting manuscripts certainly contribute to the understanding of the disease process and are escorted by remarkable work on its epidemiology. Diabetes mellitus (DM) is an increasing worldwide systemic disease and its complications, in this context, Pan et al. described a detailed picture of the association between DM and dry eye disease (DED) in Taiwan. In this retrospective population-based cohort study, they clearly identified diabetic neuropathy as a risk factor for DED in DM, which increases with the worsening of renal function in subjects with nephropathy. Additionally, they demonstrated for the first time that there is a protective effect to present DED in diabetic patients who use antihyperglycemic drugs such as DPP4 inhibitor, GLP-1 agonist, SGLT-2 inhibitor, and insulin monotherapy compared to subjects who only use metformin. Additionally, a multicenter prospective cohort study performed by Hao et al. concluded that air pollution could affect DED by different mechanisms. Exposure to particulate matter, ozone, nitrogen dioxide, and sulfur dioxide affected tear film stability and produced ocular discomfort and changes in cytokine levels. In addition, Singh et al. evaluated relationships between tear film features and age and sex in a non-DED Indian population. They found that tear meniscus height is slightly affected by age but is independent of sex, NIBUT, and TO. In this work, they propose that the notion that older people and women are at risk of present DED is less likely to be related to abnormalities in the tear film and could be associated with other different factors.

Ocular surface imaging has revolutionized diagnosis and disease monitoring but also elucidated the physiological process related to dry eye. Jing et al. demonstrated using *in vivo* confocal microscopy that the presence of oval cells and potentially immature Langerhans cells in the corneal vortex and the presence of these cell patterns were related to DED severity. Due to the complexity of measuring tear film and DED severity, Sánchez-González, Capote-Puente et al., proposed a new non-invasive method to accurately diagnose DED. The Ocular Surface Analyzer (OSA) is a device that can be adjusted to a slit lamp and is capable to measure different DED-related parameters, such as conjunctival hyperemia, meniscus height, lipid layer, NIBUT, and meibomian gland functions. The DEQ-5 items questionnaire is also included as a part of the method to make this device more accurate. Moreover, they compared in a very detailed manner its advantages and disadvantages with other methods currently used to diagnose DED and to measure tear film. OSA is proposed as a precise and practical method to be routinely used by ocular surface specialists. Garcia-Terraza et al. performed an observational study and compared three different devices for evaluating the ocular surface. They found slight differences between all three equipment types when comparing meibography images.

Dry eye treatment is rapidly changing, and novel targets and therapeutic strategies are constantly described. Yun and Min presented a study where skin temperature was evaluated before and after intense light pulsed (IPL) treatments. The authors concluded that IPL treatment decreased skin temperature, indirectly producing a reduction in superficial telangiectasia. Moreover, MGD leads to tear film instability and causes dry eye disease. Telangiectasia often coexists with MGD and is the main sign of associated posterior blepharitis. In this Research Topic, Tantipat et al. compared 0.05% bevacizumab eye drops twice a day vs. a single intra-MG injection of 2.5% bevacizumab to determine their safety and efficacy for MGD-associated posterior blepharitis. In this open-label, observer-blinded randomized controlled trial, the authors identified that both treatments are equally effective and safe for treating MGD-associated posterior blepharitis at 3 months. The authors also recommend intra-MG injection as an appropriate treatment in cases with severe lid margin telangiectasia or poor compliance with topical eye drops. DED is also a consequence of systemic drug administration like isotretinoin for acne vulgaris control, and many drugs are currently used to treat DED such as the topical administration of hyaluronic acid (HA). In this Research Topic, Sánchez-González, De-Hita-Cantalejo et al. compared the efficacy of 0.4% HA vs. 0.4% HA plus 0.2% galacto-xyloglucan (HA-GX) obtained from tamarind seed on the treatment of isotretinoin-induced DED. In this prospective, single-blinded trial, the authors demonstrated that both HA formulations are similarly effective. Moreover, HA-GX increased the BUT and mean NIBUT compared to HA alone. Keratoneuralgia treatments may vary and have different responses. Wang et al. presented a study of cases who received eye drops of autologous plasma rich in growth factors (PRGF) for keratoneuralgia or corneal nerve pain, reporting no side effects associated with this therapy. The authors concluded that PRGF treatment is safe and could improve symptoms for keratoneuralgia patients.

In summary, DED is an important health problem worldwide, and the studies published in this special edition make a significant contribution to the better understanding of this entity and improve diagnostic and therapeutic approaches for our patients. We are convinced that this Research Topic will be of interest and of great value both to scientists and clinicians. Finally, we would like to thank all the authors, institutions, reviewers and editors for their valuable time and efforts in improving this DED special report.

## Author contributions

All authors listed have made a substantial, direct, and intellectual contribution to the work and approved it for publication.

